# Identification of circulating microRNAs for the differential diagnosis of Parkinson's disease and Multiple System Atrophy

**DOI:** 10.3389/fncel.2014.00156

**Published:** 2014-06-10

**Authors:** Annamaria Vallelunga, Marco Ragusa, Stefania Di Mauro, Tommaso Iannitti, Manuela Pilleri, Roberta Biundo, Luca Weis, Cinzia Di Pietro, Angela De Iuliis, Alessandra Nicoletti, Mario Zappia, Michele Purrello, Angelo Antonini

**Affiliations:** ^1^Molecular Neurobiology Laboratory, Department for Parkinson's Disease, IRCCS Hospital San CamilloVenice, Italy; ^2^Unit of Molecular, Genome and Complex Systems BioMedicine, Department Gian Filippo Ingrassia, University of CataniaCatania, Italy; ^3^School of Biomedical Sciences, University of LeedsLeeds, UK; ^4^Department of Medicine, University of PaduaPadua, Italy; ^5^Section of Neuroscience University of Catania, Department GF Ingrassia, University of CataniaCatania, Italy

**Keywords:** Parkinson's disease, atypical parkinsonian disorders, Multiple System Atrophy, microRNAs, circulating microRNAs, early diagnosis

## Abstract

**Background:** Parkinson's disease (PD) is a progressive neurodegenerative disorder which may be misdiagnosed with atypical conditions such as Multiple System Atrophy (MSA), due to overlapping clinical features. MicroRNAs (miRNAs) are small non-coding RNAs with a key role in post-transcriptional gene regulation. We hypothesized that identification of a distinct set of circulating miRNAs (cmiRNAs) could distinguish patients affected by PD from MSA and healthy individuals. Results. Using TaqMan Low Density Array technology, we analyzed 754 miRNAs and found 9 cmiRNAs differentially expressed in PD and MSA patients compared to healthy controls. We also validated a set of 4 differentially expressed cmiRNAs in PD and MSA patients vs. controls. More specifically, miR-339-5p was downregulated, whereas miR-223^*^, miR-324-3p, and mir-24 were upregulated in both diseases. We found cmiRNAs specifically deregulated in PD (downregulation of miR-30c and miR-148b) and in MSA (upregulation of miR-148b). Finally, comparing MSA and PD, we identified 3 upregulated cmiRNAs in MSA serum (miR-24, miR-34b, miR-148b). Conclusions. Our results suggest that cmiRNA signatures discriminate PD from MSA patients and healthy controls and may be considered specific, non-invasive biomarkers for differential diagnosis.

## Background

Parkinson's disease (PD) is a neurodegenerative disorder characterized by rigidity, tremor, bradykinesia, and postural instability. Clinical diagnosis is challenging and misdiagnosis rate is comprised between 10 and 30% in early stages mainly due to failure to recognize atypical parkinsonism (Poewe and Wenning, 2002). The commonest atypical form is Multiple System Atrophy (MSA), a sporadic neurodegenerative disorder characterized by neuronal cell loss and gliosis in specific brain areas including the basal ganglia (Paik et al., 2010). Early differentiation between PD and MSA has clinical, therapeutic and prognostic consequences and may be difficult, if based solely on clinical examination (Galvin et al., 2001; Campbell and Choy, 2012). Specific imaging abnormalities can be present but only when the symptoms are fully established (Ghaemi et al., 2002), while identification of disease-specific and minimally invasive biomarkers would be required for early diagnosis. Recent evidence suggests that the post-transcriptional regulation is an important process in PD pathophysiology (Imai et al., 2008; Blackinton et al., 2009; Tain et al., 2009; Harraz et al., 2011). MicroRNAs (miRNAs) are small non-coding RNAs, which have been identified as post-transcriptional regulators of gene expression (Graves and Zeng, 2012); miRNAs can inhibit protein coding genes by affecting mRNA translation and/or stability. MiRNAs play important regulatory roles in many cellular processes as cell replication, differentiation and neoplastic transformation (Etheridge et al., 2011; Ragusa et al., 2012) and in granting the survival of mature neurons and their functions (Hong et al., 2013). Recent studies have shown that some miRNAs are differentially expressed (DE) in human brain and may regulate the expression of genes associated with Alzheimer's disease (AD) and PD (Schonrock et al., 2010; Miñones-Moyano et al., 2011; Bekris et al., 2013; Cho et al., 2013; Lau et al., 2013). PD with and without dementia revealed lower levels of miR-205 in the frontal cortex and striatum, if compared to healthy controls (Cho et al., 2013), while another study showed that miR-34b and mir34c are downregulated in several PD brain areas (Miñones-Moyano et al., 2011). A signature of 18 miRNAs under-expressed in PD peripheral blood mononuclear cells has been suggested to distinguish patients from controls (Martins et al., 2011). Other studies have identified specific miRNAs targeting and down-regulating the expression of PD-related genes and demonstrating a reciprocal relationship between PD-related genes and miRNA processing (Heman-Ackah et al., 2013). Junn et al. showed higher miR-7 levels in the substantia nigra and striatum of mice compared to cerebral cortex and cerebellum. MiR-7 levels were found to be 40 times higher in neurons than in astrocytes (Junn et al., 2009). Moreover alpha synuclein was detected in neurons, but not in astrocytes (Junn et al., 2009). Doxakis et al. described a similar alpha synuclein regulation by miR-7 and miR-153 (Doxakis, 2003). Recent studies have also reported deregulated miRNAs levels in PD patients plasma compared to normal subjects (Khoo et al., 2012; Cardo et al., 2013). Circulating miRNAs (cmiRNAs) possess many key features typical of reliable biomarkers, since they are easily detectable, stable in many body fluids, and easily measurable by PCR. Finally, alterations of several cmiRNA levels in plasma, serum, urine, and saliva have been associated with different diseases, as acute myocardial infarction, congestive heart failure, different types of cancer, and multiple sclerosis (Etheridge et al., 2011). A panel of serum cmiRNAs (mir-29, mir-29c, mir-19a/b) were downregulated in carriers of LRKK2 G2019S mutation and in idiopathic PD patients compared to healthy controls (Botta-Orfila et al., 2014).

In this study, for the first time, we tested the hypothesis that specific panels of cmiRNAs could differentiate PD from MSA patients and represent a potential biomarker to be applied in clinical setting.

## Materials and methods

### Patients and sample collection

Study participants were recruited from the Parkinson Unit at the San Camillo Hospital (Venice, Italy) and the 1st Neurology Clinic at the University Hospital of Padua (Padua, Italy). In the discovery set, we enrolled six patients affected by PD (55 ± 5.5 years mean ± s.e.m.), six patients affected by MSA, parkinsonian subtype P (MSA-P) (63± 10 years mean ± s.e.m.), and three patients affected by MSA, cerebellar dysfunction subtype C (MSA-C) (62 ± 2 years mean ± s.e.m.). Healthy controls included five age- and ethnicity-matched subjects (59 ± 3.08 years mean ± s.e.m.; range = 55–63), with no history of neurological or psychiatric diseases. Furthermore, we excluded subjects with inflammatory and systemic diseases, as diabetes and other cardiovascular diseases. In the validation set, we enrolled 75 subjects: 25 PD, 25 MSA and 25 healthy controls. All PD patients were diagnosed according to the UK Brain Bank Criteria (Hughes et al., 1992) Clinical diagnosis was confirmed by two independent and certified neurologists. Inclusion criteria were: disease duration of less than 6 years from diagnosis, mild to moderate disease stage (Hoehn and Yahr score > 2.5), no significant cognitive deficit (Mini Mental State Examination (MMSE); score > 26) and age between 46 and 60 years (Hoehn and Yahr, 1967; Folstein et al., 1975). All MSA patients were diagnosed according to Gilman's criteria and the diagnosis was confirmed by two independent certified neurologists (Gilman et al., 1998). We included MSA patients over the age of 50 and clinical severity was evaluated with the Unified Multiple System Atrophy Rating Scale (Wenning et al., 2004). Exclusion criteria for the selection of PD and MSA patients were the same and included: presence of other neurological disorders, PD and neurodegenerative disease familiarity, clinical dementia (based on DSM-IV criteria), early and late disease onset, presence of concomitant systemic diseases, as diabetes and other cardiovascular diseases and MMSE < 26 corrected for age and education. The demographic data (age and gender) and neurological details are shown in Table 1. All patients had been tested for the G2019S-LRKK2 and PARK2 and SNCA mutations and none of those included in this study was homozygous or heterozygous for these mutations. All enrolled patients underwent fasting venous blood sampling. Blood samples were obtained by vein puncture using dry vacutainer tubes (BD Biosciences, Italy). The blood samples were processed by the Laboratory of Molecular Biology of IRCCS San Camillo. The samples were processed for serum isolation within 2 h after withdrawal. Whole blood was left to stand for about 30′ at 20°C before being centrifuged at 3000 rpm for 15′ at 4°C. Serum was divided into aliquots, and stored at -80°C until analysis.

**Table 1:** **Clinical characteristics of patients and healthy controls**.

**Characteristics**	**PD**	**MSA**	**Healthy controls**
N	25	25	25
Male/Female	13 (12)	12 (13)	13 (12)
UPDRS	37.05 (9.8)	37.88 (12.88)	–
LDED	730.48 (392.8)	708.27 (319.35)	–
Dopamine agonist	126.7 (127)	75 (82.11)	–
H and Y	3 (0.03)	3 (0.08)	–

*LDED, L-dopa daily equivalent dose; H and Y, Hoehn and Yahr scale*.

#### RNA isolation, reverse transcription, and miRNA profiling by TaqMan Low Density Array

Serum samples were centrifuged at 2000 rpm for 10′ to pellet and remove any circulating cell or debris. miRNAs were extracted from 400 μl of serum samples by using Qiagen miRNeasy mini kit (Qiagen, GmbH, Hilden, Germany), according to Qiagen supplementary protocol for purification of small RNAs from serum and plasma, and finally eluted in 30 μl volume of elution buffer (Ragusa et al., 2013). RNAs were quantified by fluorometer and spectrophotometer. To profile the transcriptome of 754 miRNAs, 3.2 μl of serum RNAs (corresponding to about 20 ng of RNA) were retrotranscribed and pre-amplified, according to the manufacturer's instructions. Pre-amplified products were loaded onto TLDAs, TaqMan Human MicroRNA Array v3.0 A and B (Applied Biosystems | Life Technologies™ Monza, Italy). PCRs on TLDAs were performed on 7900HT Fast Real Time PCR System (Applied Biosystem | Life Technologies™ Monza, Italy). Result validation was obtained by single TaqMan assays (Applied Biosystems | Life Technologies™ Monza, Italy) using the same amount of serum miRNAs according to the manufacturer's instructions.

### Data analysis

To obtain an accurate miRNA profiling, we used the global median normalization method. Similarly to microarray analysis, Ct values from each sample were normalized to the median Ct of the array (Ragusa et al., 2010). Moreover, by computing the Pearson correlation among the Ct medians and means of each array and Ct of each miRNA, we identified two miRNAs that showed an expression profile closer to the median and mean of TLDAs, i.e., miR-17 and miR-151-3p. These miRNAs also resulted among the most stable in TLDAs by applying two different methods [DataAssist v.3 software (Applied Biosystems | Life Technologies™ Monza, Italy)] and geNorm Algorithm (http://medgen.ugent.be/~jvdesomp/genorm/). Accordingly, miR-17 and miR-151-3p were used as reference genes for validation by single TaqMan assays. Expression fold changes were calculated by the 2^−ΔΔ*CT*^ method. DE miRNAs were identified by Significance of Microarrays Analysis (SAM) computed by Multi experiment viewer v4.8.1 (http://www.tm4.org), applying a two-class unpaired test among ΔCts and using a *p*-value based on 100 permutations and imputation engine: K-nearest neighbors (10 neighbors). False discovery rate<0.15 was used as correction for multiple comparisons. We accepted as reliable only DE miRNAs concordant by using all endogenous controls. Expression data in “Results” are shown as average relative quantity (RQ) of all RQ values, calculated with each endogenous control respect to normal controls. RQ values < 1 were converted to negative fold changes by following the formula: -1/RQ. The Wilcoxon signed-rank test (*p* < 0.05) was applied to statistically evaluate expression differences between patients affected by PD or MSA and healthy controls in single TaqMan validation assays.

### miRNA target prediction

In order to increase data strength, DE miRNA targets were analyzed by a combination of two different approaches. By interpolating 11 prediction tools (http://mirecords.biolead.org), a first series of predicted and experimentally validated DE miRNA targets was extracted from miRecords. To improve our prediction, an additional filtering was performed by using starBase, a database for predicted miRNA–target interactions, overlapped with data from Argonaute cross-linked immunoprecipitation sequencing (CLIP-Seq) (Yang et al., 2011). CLIP-Seq experiments are based on crosslinking between RNA and proteins, followed by immunoprecipitation coupled to high-throughput sequencing. The application of this technique is applied to identify miRNA binding sites.

### Gene ontology analysis

The identification of statistically significant Gene Ontologies of miRNA targets was obtained by using FatiGo (Biological Process) from Babelomics 4.2 server (http://babelomics.bioinfo.cipf.es/). We used the gene functional classification tool DAVID (http://niaid.abcc.ncifcrf.gov) to identify tissue-specific expression of miRNA targets.

#### Ethics statement

This study was conducted according to the Declaration of Helsinki and was approved by the ethics committee of IRCCS San Camillo, Venice (Italy). A written informed consent was obtained from all the patients participating in this study.

## Results

### Expression profiles by TaqMan Low Density Arrays

To determine whether there was a unique PD- and MSA-associated miRNA profile, which could be identified in serum, we first tested 20 samples (discovery set) using TaqMan Low Density Array technology. We initially determined the expression profile of 754 miRNAs in serum of 6 PD patients and 9 MSA (6 with MSA-P and 3 subjects affected by MSA-C). These serum profiles were compared with those of five normal subjects. From 754 screenable miRNAs of TLDA, we identified 324 circulating miRNAs in all our serum samples. Delta Cts obtained from these profiles are shown in complementary data 1 and graphically plotted in complementary data 2. By applying SAM method, we highlighted differentially expressed (DE) miRNAs by performing four comparisons among these miRNA profiles: (a) whole cohort of pathological samples (MSA + PD) vs. the healthy ones; (b) PD samples vs. controls; (c) MSA samples vs. controls; MSA samples vs. PD samples. We found 8 DE miRNAs (3 downregulated, 5 upregulated) from the first comparison; 9 DE miRNAs from the second comparison (4 downregulated, 5 upregulated); 12 DE miRNAs from the third comparison (3 downregulated, 9 upregulated); 5 DE miRNAs from MSA vs. PD comparison (1 downregulated, 4 upregulated). These data are reported in Table 2.

**Table 2:** **DE MiRNAs in PD and MSA patients compared to healthy controls**.

**MSA+PD vs. CTRL**		**PD vs. CTRL**		**MSA vs. CTRL**		**MSA vs. PD**	
**DE miRNAs (TLDA)**	**Average FC (single assay) (*p*-value)**	**DE miRNAs (TLDA)**	**Average FC (single assay) (*p*-value)**	**DE miRNAs (TLDA)**	**Average FC (single assay) (*p*-value)**	**DE miRNAs (TLDA)**	**Average FC (single assay) (*p*-value)**
**mir-24**	**7.2 (0.00004)**	**mir-24**	**2.94 (0.03)**	**miR-24**	**4.45 (0.00008)**	**miR-24**	**2.35 (0.00004)**
mir-34b	1.59 (0.375)	**miR-30c**	**−1.53 (0.036)**	mir-29c	1.22 (0.102)	**miR-34b**	**3.9 (0.012)**
miR-223^*^	**2.31 (0.001)**	miR-34b	1.76 (0.07)	**miR-148b**	**1.78 (0.00009)**	**miR-148b**	**3.19 (0.0006)**
**miR-324-3p**	**3.29 (0.0001)**	**miR-148b**	**−1.53 (0.039)**	mir-223^*^	**3.35 (0.0003)**	miR-339-5p	1.34 (0.34)
**miR-339-5p**	**−2.57 (0.007)**	miR-223^*^	**2.09 (0.006)**	**miR-324-3p**	**4.04 (0.0002)**	mir-1274A	**−**1.12 (0.485)
mir-652	**−**1.29 (0.202)	**miR-324-3p**	**1.89 (0.036)**	**miR-339-5p**	**−1.64 (0.032)**		
mir-744	**−**1.53 (0.378)	mir-339-5p	**−**1.88 (0.358)	mir-483-5p	1.16 (0.407)		
miR-1274A	3.42 (0.31)	mir-652	**−**1.63 (0.21)	mir-652	**−**1.53 (0.244)		
		miR-1274A	4.34 (0.12)	mir-744	**−**1.43 (0.402)		
				miR-1274A	2.45 (0.09)		
				mir-1274B	1.89 (0.086)		
				miR-1291	4.45 (0.052)		

*Differentially Expressed miRNAs identified in this study by TLDAs and their validation by single TaqMan assays. DE, Differentially Expressed; FC, Fold Change. MiRNAs statistically validated by single assays are typed in bold text*.

* *Part of the name of the microRNA according with microRNA nomenclature*.

### Differential expression of miRNAs among PD and MSA patients and healthy controls

**Figure 1:**
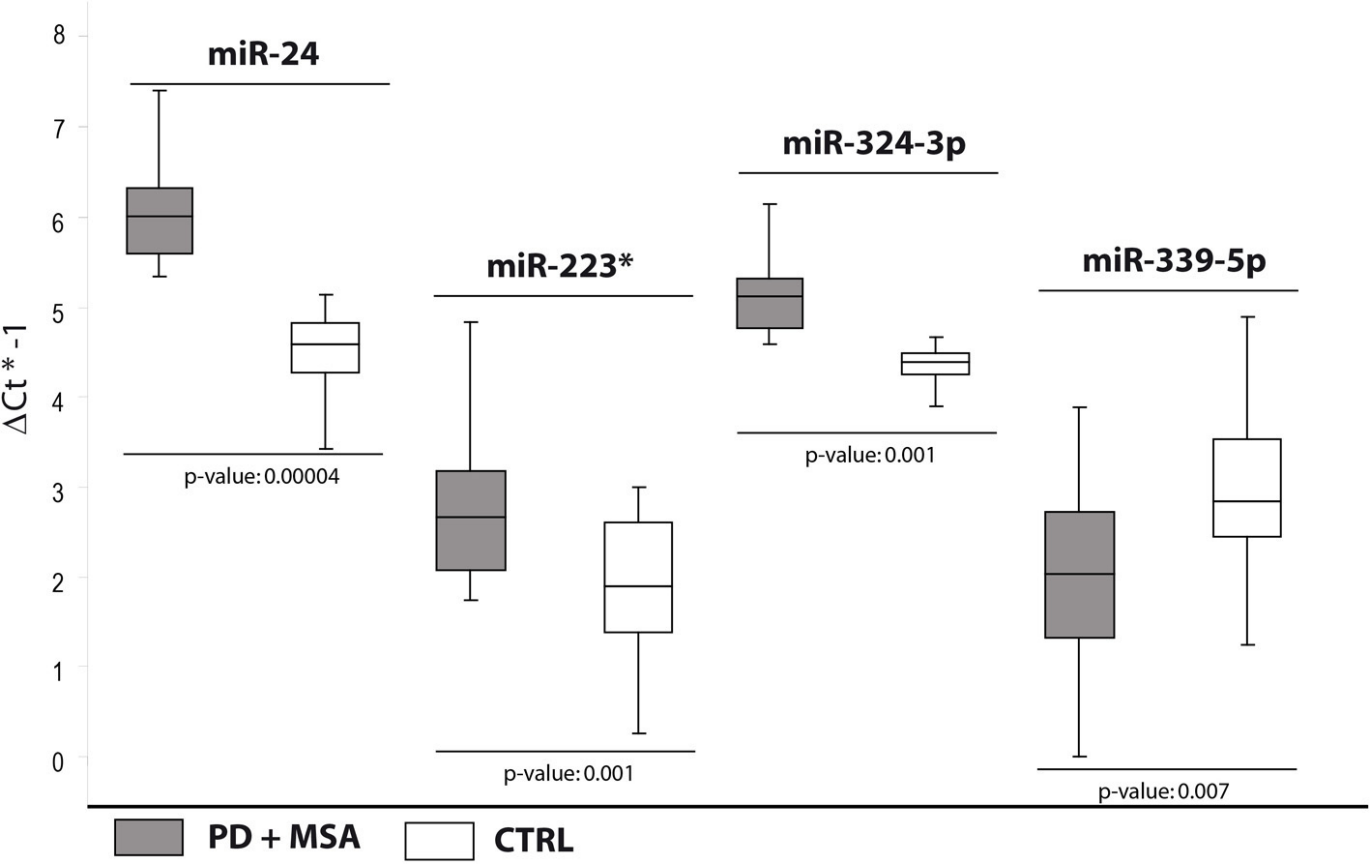
**Box and Whisker plot of DE cmiRNAs validated after single TaqMan assays in PD and MSA patients compared to controls**. Values on the y-axis are reported as ΔCt × (−1). White boxes: healthy controls; gray boxes: PD and MSA patients. The bottom and top of the box are the first and third quartiles; the band inside the box is the second quartile (the median); the ends of the whiskers represents minimum and maximum values of the data. Samples analysed: 30 CTRL; 31 PD + 34 MSA. Statistical significance was evaluated by the Wilcoxon rank sum test (*p* < 0.05). ^*^Part of the name of the microRNA according with microRNA nomenclature.

In order to validate these findings, we subsequently tested the expression of DE miRNAs in the same samples analyzed by TLDA and in a separate and independent cohort of patients (25 PD and 25 MSA) and 25 healthy controls by using single TaqMan assays and applying the Wilcoxon rank-sum test (*p* < 0.05). Data validation by single TaqMan assays resulted in identification of upregulation for miR-24, miR-223^*^, miR-324-3p and downregulation for miR-339-5p (Table 2, Figure 1).

### Specific dysregulation of cmiRNAs in PD patients compared with healthy controls

**Figure 2:**
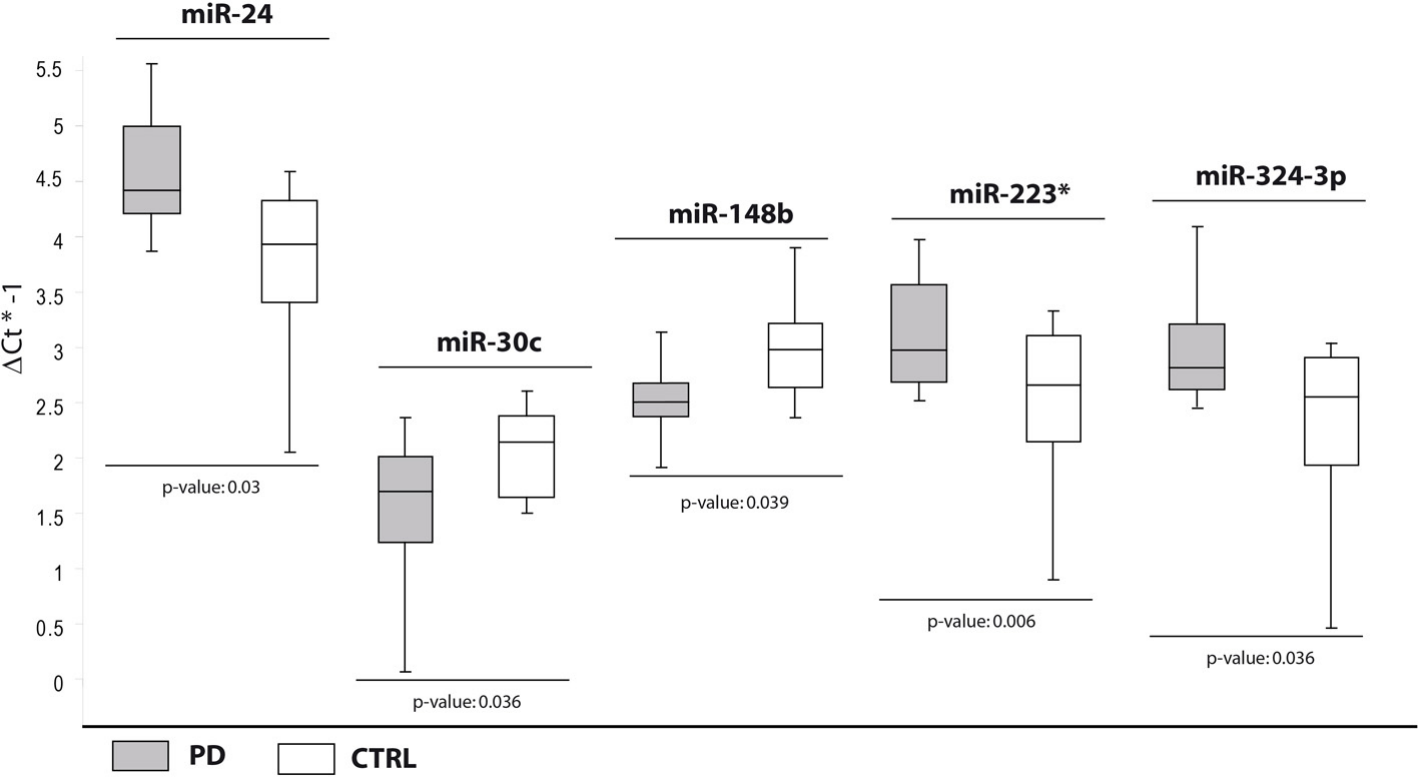
**Box and Whisker plot of DE cmiRNAs validated after single TaqMan assays in PD patients compared with healthy controls**. Values on the y-axis are reported as ΔCt × (−1). White boxes: healthy subjects; light gray boxes: PD patients. Samples analysed: 30 CTRL; 31 PD. Statistical significance was evaluated by the Wilcoxon rank sum test (*p* < 0.05). ^*^Part of the name of the microRNA according with microRNA nomenclature.

In the second comparison, we pinpointed DE miRNAs specific for PD (Table 2). We initially identified nine DE miRNAs by high throughput profiling, but only five were successfully validated: miR-24, miR-223^*^, miR-324-3p resulted upregulated in PD patients with respect to healthy controls, while miR-30c and miR-148b were downregulated (Table 2, Figure 2). The downregulation of miR-30c and miR-148b in PD patients compared to healthy controls seemed to be specific for PD.

### cmiRNAs differentially expressed between MSA patients and healthy controls

**Figure 3:**
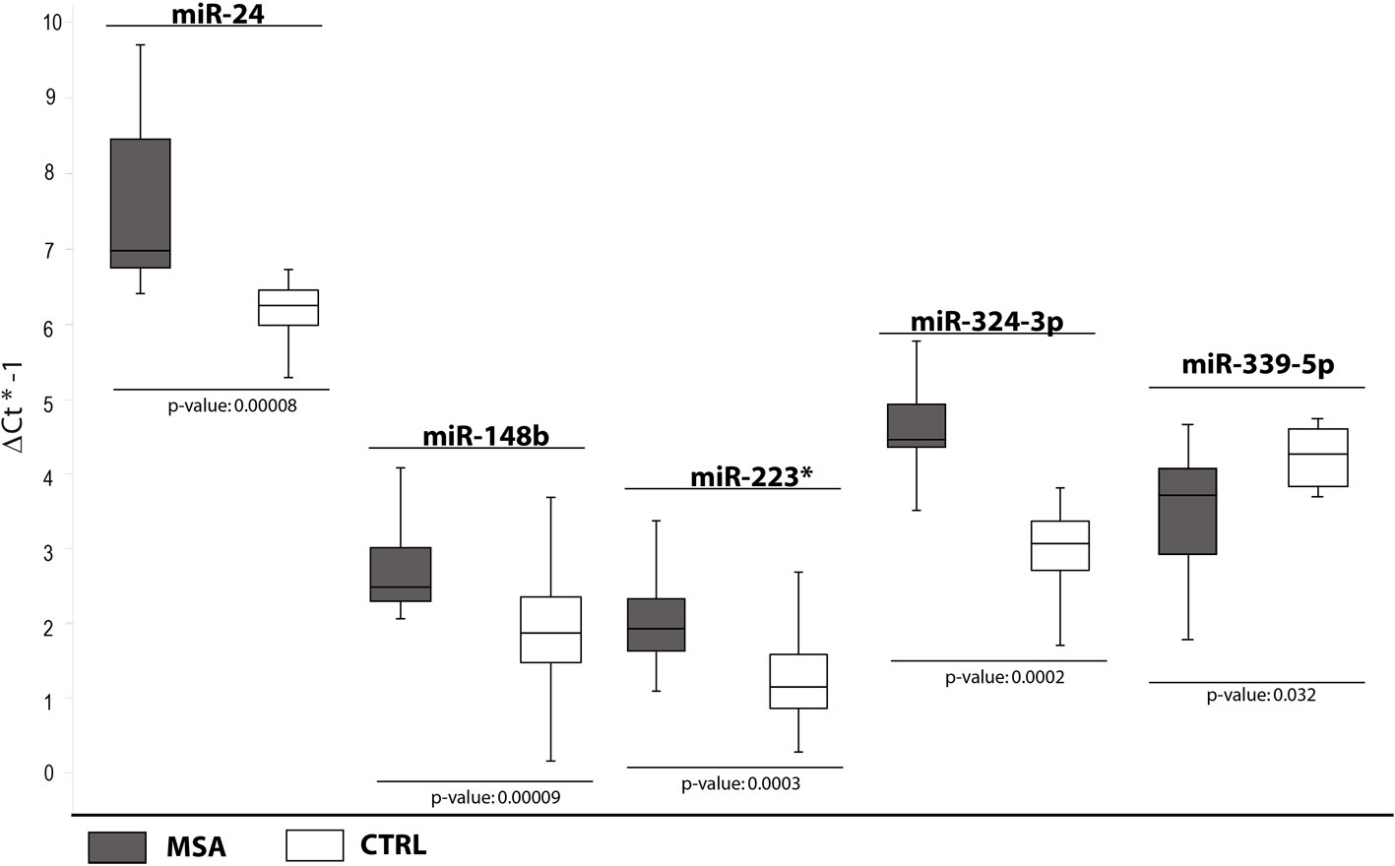
**Box and Whisker plot of DE cmiRNAs validated after single TaqMan assays in MSA patients compared to healthy controls**. Values on the y-axis are reported as ΔCt × (−1). White boxes: healthy patients; dark gray boxes: MSA patients. Samples analysed: 30 CTRL; 34 MSA. Statistical significance was evaluated by the Wilcoxon rank sum test (*p* < 0.05). ^*^Part of the name of the microRNA according with microRNA nomenclature.

In the third comparison, we identified DE miRNAs specific for MSA patients: the initial set of 12 DE miRNAs was further filtered by single assay validation. This confirmed overexpression of miR-24, miR-148b, miR-223^*^, miR-324-3p, and downmodulation of miR-339-5p (Table 2, Figure 3). Interestingly, upregulation of miR-24, miR-223^*^, and miR-324-3p was significant in PD and MSA subjects compared with healthy controls, both grouped together or analyzed as specific disease groups. These data suggest that deregulation of these circulating miRNAs could originate from pathological features common to both diseases.

It is interesting to note that downregulation of miR-339-5p was detected for PD + MSA vs. controls (Ctrl) Unexpectedly, following the specific-disease analysis, this miRNA was differentially expressed only in MSA vs. Ctrl. This statistical ambiguity may be due to the homogenization of expression data by mixing different pathological groups, although phenotypically similar.

### miRNAs differentially expressed in PD and MSA patients

**Figure 4:**
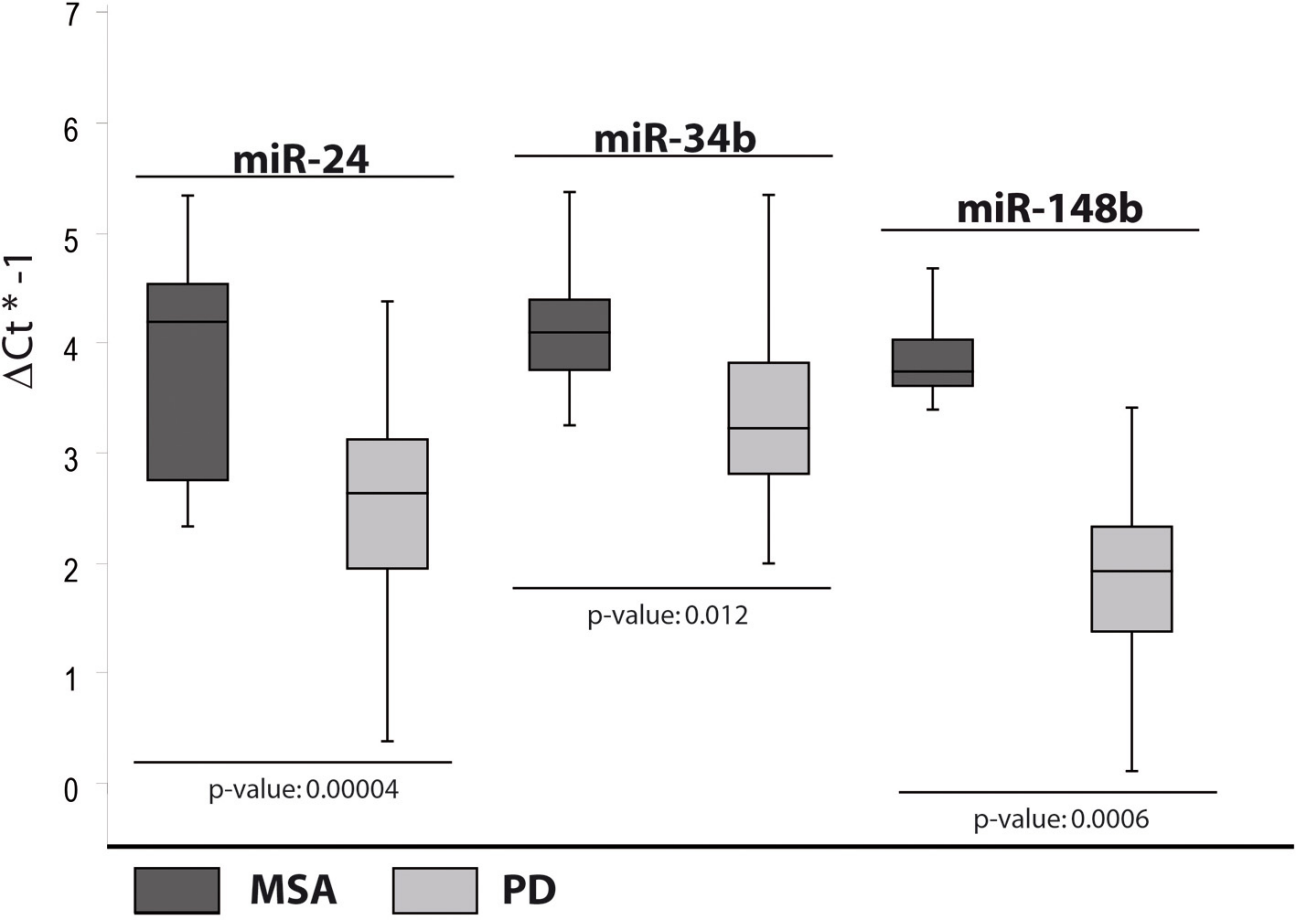
**Box and Whisker plot of DE cmiRNAs validated after single TaqMan assays in PD patients compared to MSA patients**. Values on the y-axis are reported as ΔCt × (−1). Light gray boxes: PD patients; dark gray boxes: MSA patients. Samples analysed: 34 MSA; 31 PD. Statistical significance was evaluated by the Wilcoxon rank sum test (*p* < 0.05).

In the fourth analysis, we directly compared the cmiRNA transcriptome of MSA patients with that of PD patients in search of discriminating miRNAs between the two pathological phenotypes. Statistical analysis of whole miRNA profiles initially showed five DE miRNAs. We confirmed the deregulation of three of them through single Taqman assays: miR-24, miR-34b, and miR-148b were upregulated in serum of MSA patients with respect to PD patients (Table 2, Figure 4).

On the other hand, upregulation of miR-148b, observed only in MSA patients if compared to PD subjects, seemed to be a valid discriminating factor.

### miRNA targets and gene ontology analysis

**Figure 5:**
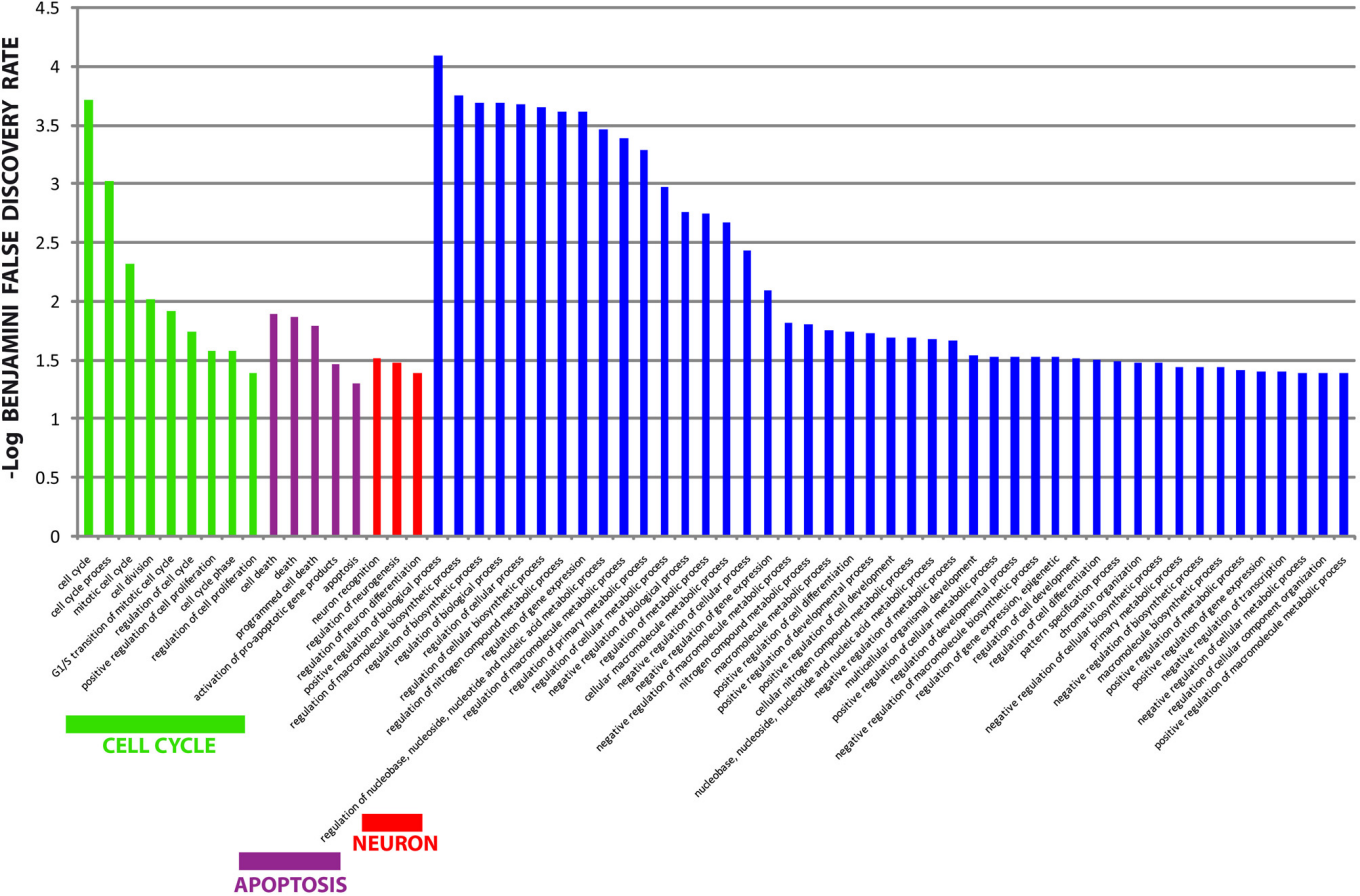
**Gene Ontology analysis of targets from differentially expressed miRNAs**. Overrepresented biological processes of miRNA targets. Data are shown as -log10 of Benjamini and Hochberg false discovery rate for each term. The Blue bars showed non-informative biological terms (too generic terms or unrelated terms).

**Figure 6:**
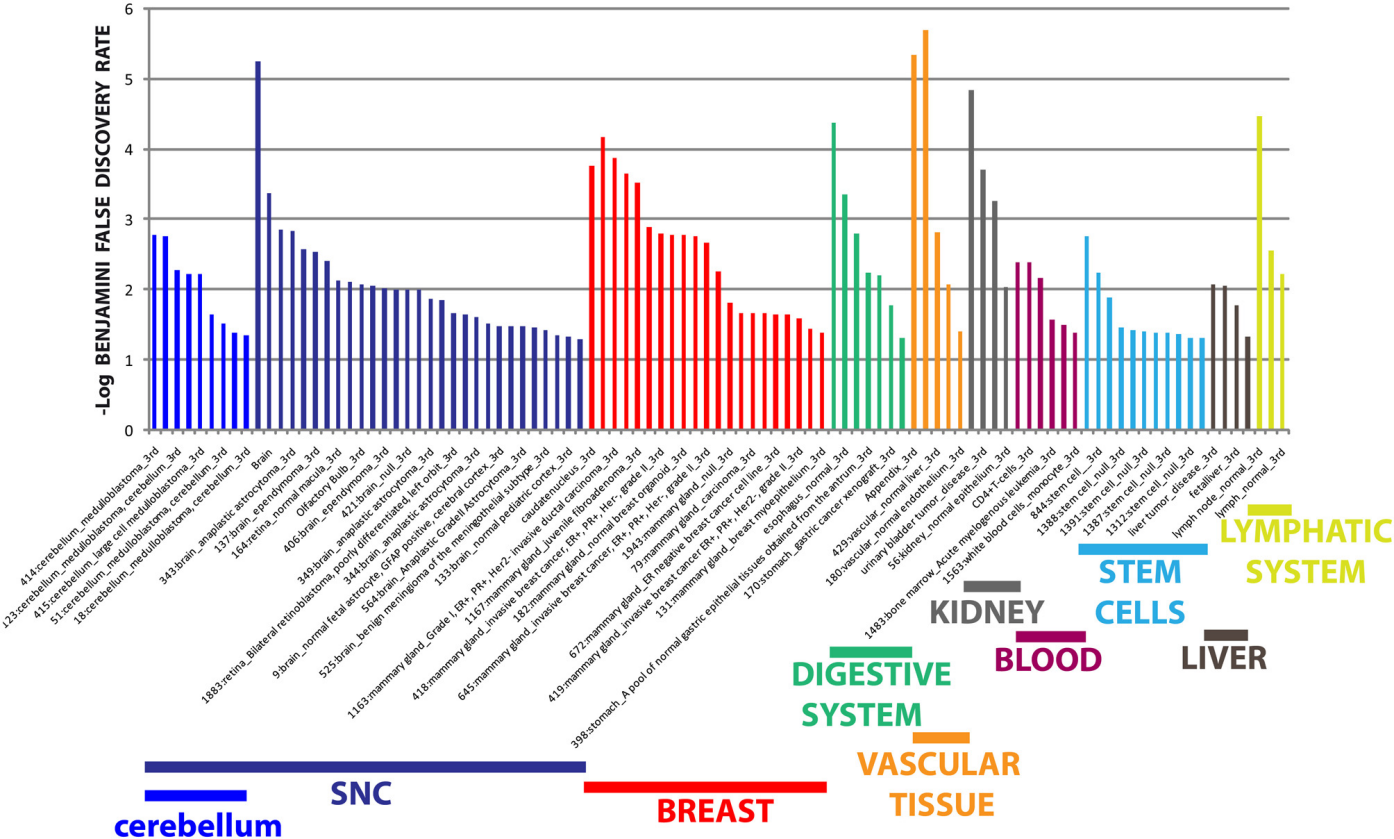
**Tissue expression analysis of targets from differentially expressed miRNAs**. Tissue-specific overexpression of miRNA targets. Data are shown as -log10 of Benjamini and Hochberg false discovery rate for each term.

The different distribution of these miRNAs in serum from PD and MSA patients is most likely a systemic consequence of the different physiopathology of these two diseases. In order to evaluate the biological functions of DE miRNAs, we computationally searched their validated or predicted targets as shown above. Gene ontologies of miRNA targets were analyzed by FatiGo. This analysis showed that they are involved in important biological processes of Parkinson and Parkinsonism physiopathology, as cell cycle regulation, modulation of apoptosis and post-translational modifications (Figure 5). Moreover, by using the gene functional classification tool DAVID, we observed that the targets of DE miRNAs were mainly expressed in the central nervous system (CNS) and in other tissues as breast, vascular and digestive systems and kidney (Figure 6). Taken together, these data suggest that the circulating DE miRNAs, which we identified, derive from tissues typically involved in PD and MSA and could be involved in dysfunctional biological processes of the two diseases.

## Discussion

Our study provides the first report of whole serum miRNAs transcriptome profiling in PD and MSA. First, we conducted a comprehensive screening of miRNAs expression using a TaqMan Low density Arrays (TLDAs) and, after that, we performed single RT-qPCR assays on a second patient dataset to independently confirm miRNAs profile data. Our analysis identified eight miRNAs differentially expressed in PD and MSA vs. controls and in PD vs. MSA. Mir-324-3p, miR-24 and miR-223^*^ were identified in the largest number of comparisons. Our results expand findings from previous studies analyzing miRNAs levels in blood from PD. All serum cmiRNAs previously implicated in PD (Khoo et al., 2012; Cardo et al., 2013; Botta-Orfila et al., 2014) were expressed at detectable levels in our samples, but no significant statistical differences were observed. Probably, those differences were related to different inclusion criteria used in our study. For example, we included patients with less than 6 years' disease duration from diagnosis or mild-to-moderate disease stage (Hoehn and Yahr score > 2.5), while in the study by Botta-Orfila et al., Hoehn and Yahr score was 2 and disease duration was higher. A growing body of evidence shows that miRNA expression changes with age and also the serum levels of specific miRNAs change markedly. Otherwise, only a few plasma cmiRNAs previously implicated in PD (Khoo et al., 2012; Cardo et al., 2013) were expressed at detectable levels in PD and MSA samples but their differences were not statistically significant. miRNAs expression pattern in brain, blood, sputum and urine may be a diagnostic and prognostic biomarker (Ezzie et al., 2012).

Discrepancies in miRNA expression reported in other studies may result from technical (e.g., sample preparation) as well as biological (e.g., tissue) differences. In our study, we focused on serum not only because it has practical advantages, but also because it is free of anticoagulants as heparin, (a potent inhibitor of PCR reactions) (Yokota et al., 1999). It is also important to note that patient blood and serum may be less affected by haemolysis, if compared with plasma samples. A recent study suggests a contribution by blood-cell specific and platelet specific microRNAs deriving from plasma haemolysis and resulting in significant variation of microRNA expression (Pritchard et al., 2012). Inadequate handling of plasma samples could increase the risk of hemolysis and dilute the quality of downstream data analysis. By contrast, the standard serum isolation procedure is relatively more consistent as whole blood samples are set to coagulate naturally. Thus, data comparison of serum samples collected at different research centers would be more reliable (Li and Kowdley, 2012).

### Deregulation of circulating miRNAs in synucleinopathies

We observed that the miR-324-3p was upregulated in PD and MSA patients vs. healthy controls. Liu et al. showed that miR-324-3p was downregulated in the brain of an embolic stroke model and its expression may be an indicator of recovery (Liu et al., 2013). In addition, Stappert et al. observed that this miRNA was upregulated in neural cells compared to human embryonic stem cells and further increased upon differentiation (Smirnova et al., 2005; Stappert et al., 2013). Several studies indicated that miR-324-3p is underexpressed in brain tumor cells and suggested its hypothetical role as tumor suppressor (Smirnova et al., 2005; Ferretti et al., 2008; Stappert et al., 2013). In our study, miR-223^*^ was upregulated in PD and MSA patients vs. healthy controls. To date, there are no data on the physiological and pathological role of miR-223^*^, but there are several reports on the biological functions of its sister miRNA, miR-223. Izumi et al. reported an increase in miR-223 expression after spinal cord injury and indicated that it could regulate neutrophils in the early phase after lesion (Izumi et al., 2011). Harraz et al. showed that miR-223 controls the response to neuronal injury by regulating the functional expression of the glutamate receptor subunits GluR2 and NR2B in the brain. However, this study revealed a unique function for miR-223 in the CNS by targeting the AMPAR subunit of GluR2 and the NMDAR subunit of NR2B which, in turn, control neuronal excitability in response to glutamate (Harraz et al., 2012). We observed that miR-24 was upregulated in PD and MSA patients vs. healthy controls and in MSA vs. PD patients. Dutta et al. showed that miR-24 is upregulated in demyelinated hippocampi from post-mortem multiple sclerosis brains. They also found that miR-24 is expressed in mouse hippocampal neurons (Dutta et al., 2013) and cardiac endothelial cells. MiR-24 is also considerably upregulated following myocardial ischemia (Zhu and Fan, 2012).

### cmiRNA dysregulation in PD patients

miR-30c was downregulated only in PD patients compared to healthy controls. Meder et al. showed that blood miR-30c was upregulated and correlated with infarct sizes (Meder et al., 2011). A study showed that miR-148b was downregulated in human AD brain, specifically in the parietal lobe cortex (Nunez-Iglesias et al., 2010), while another revealed downregulation in both hippocampus and medial frontal gyrus, but not in cerebellum from AD patients (Cogswell et al., 2008). In addition, Zhou et al. demonstrated that lithium and valproate regulate miR-30c expression in primary hippocampal neuronal cultures (Zhou et al., 2009).

### cmiRNAs dysregulation in MSA patients

Only mir-339-5p was specifically downregulated in MSA, but not in PD compared with controls. However, mir-339-5p was also downregulated when PD and MSA patients were pooled together and compared to healthy controls. This miRNA is downregulated in mature neurons and associated with the important process of axon guidance (Liu et al., 2012). Interestingly, miR-339-5p was also identified as endogenous control for normalization of miRNAs in primary medulloblastoma and human neural stem cells (Genovesi et al., 2012).

### Deregulated cmiRNAs in MSA patients in comparison with PD subjects

In our study, we showed that expression levels of miR-24, miR-34b, and miR-148b were increased in serum from MSA patients compared to PD subject. Gaughwin et al. showed that miR-34b was significantly elevated in plasma from Huntington's disease gene carriers prior to symptoms onset (Gaughwin et al., 2011). A recent microRNA profiling study in brains from PD patients revealed a significant miR-34b decrease (40–65% compared to controls) in affected brain regions, e.g., amygdala, substantia nigra, and frontal cortex, also showing a modest trend in the cerebellum. Significant reductions in this miRNA were also detected in the amygdala, but not in the frontal cortex of patients with incidental Lewy body disease, a presumed pre-motor stage of PD. Since these individuals were asymptomatic during their life and had not received anti-parkinsonian therapies, these alterations may be part of the process occurring in the early phases of the disease, rather than secondary to therapeutic interventions (Miñones-Moyano et al., 2011).

### Circulating miRNAs as potential biomarkers for PD

To date, the presence of miRNAs in biological fluids represents a subject of considerable debate. The most accepted hypothesis proposes that miRNAs are actively secreted in membrane-bound vesicles (e.g., exosomes, microvesicles) (Valadi et al., 2007). However, recent studies have shown that the majority of cmiRNAs are present in plasma and serum in a non-membrane-bound form, protected by complexes comprising proteins of the Argo family (Wang et al., 2010). Moreover, the hypothesis that cmiRNAs would be by-products of dead cells is not to be completely excluded. During the inflammatory processes or apoptosis, neurons could reciprocally exchange molecular signals through miRNAs or proteins secreted in cerebrospinal fluid or blood exosomes (Russo et al., 2012; Chang et al., 2013). This horizontal molecular transfer could also occur between brain and distant organs via biological fluids. A presumed mechanism involves the transcytosis of exosomes across endothelial cells of the blood–brain barrier by receptor-mediated endocytosis and release of the exosomal cargo into systemic circulation (Haqqani et al., 2013). Accordingly, cmiRNAs detected in serum or plasma may be biomarkers reflecting pathological brain status. Notably, the functional classification of protein targets of the miRNAs analyzed in our work showed an overabundance of terms related to CNS, suggesting that the brain could be the main source of DE miRNAs.

## Conclusions

This study is the first global miRNAs expression analysis performed in serum of PD and MSA patients. As the effective cellular function of circulating miRNAs remains mostly unknown and the molecular and phenotypic complexity of PD linger to unravel, miRNA studies could shed light on its molecular pathogenesis and possibly serve as disease specific biomarkers. Future prospective trials on larger cohorts are warranted to confirm whether this set of miRNAs can be effectively used for early PD and MSA diagnosis.

## Complementary data 1

Table of Delta Ct values obtained from TLDA profiling in serum from MSA, PD and control patients. Delta Cts were calculated by normalizing Ct values to median of each TLDA. All miRNAs not detectable by TLDA were excluded from calculation.

## Complementary data 2

Expression matrix of 324 miRNAs detectable by TLDA in serum from MSA, PD, and control patients. Data are shown as Delta Ct normalized to median of TLDAs. Rows, miRNA; columns, samples. Delta Ct are shown according to the colored bar shown below the matrix.

## Author contributions

Annamaria Vallelunga and Marco Ragusa conceived and performed the experiments, were involved in the acquisition, analysis and interpretation of the data. Annamaria Vallelunga, Marco Ragusa and Tommaso Iannitti wrote the manuscript. Cinzia Di Pietro, Stefania Di Mauro, Angela De Iuliis, Tommaso Iannitti, Luca Weis, Alessandra Nicoletti, Roberta Biundo and Manuela Pilleri were involved in the design of the work, in the acquisition, in the analysis and interpretation of the data. Mario Zappia Angelo Antonini and Michele Purrello were involved in the design, of the study, wrote and revised the article critically for important intellectual content, have given final approval of the version to be published. All authors read and approved the final manuscript.

### Conflict of interest statement

The authors declare that the research was conducted in the absence of any commercial or financial relationships that could be construed as a potential conflict of interest.
